# The Oxygen Reduction Electrocatalytic Activity of Cobalt and Nitrogen Co-doped Carbon Nanocatalyst Synthesized by a Flat Template

**DOI:** 10.1186/s11671-016-1804-z

**Published:** 2017-02-22

**Authors:** Chaozhong Guo, Youcheng Wu, Zhongbin Li, Wenli Liao, Lingtao Sun, Chao Wang, Bixia Wen, Yanrong Li, Changguo Chen

**Affiliations:** 1 Research Institute for New Materials Technology, School of Materials and Chemical Engineering, Chongqing University of Arts and Sciences, Chongqing, 402160 China; 2 School of Chemistry and Chemical Engineering, Chongqing University, Chongqing, 400044 China

**Keywords:** Oxygen reduction, Catalyst, Polyaniline, Montmorillonite

## Abstract

The design of noble-metal-free catalysts for oxygen reduction reaction (ORR) is very important to the commercialization of fuel cells. Here, we use a Co-modified montmorillonite (Co-MMT) as a flat template to prepare Co- and N-doped nanocarbon ORR catalysts derived from carbonization of polyaniline at controlled temperatures. The use of flat template can hinder the agglomeration of polyaniline during pyrolysis process and optimize the N-rich active site density on the surface. The addition of transition metal Co in the flat MMT template can largely promote the formation of Co–N sites in prepared catalyst, facilitating the effective improvement of catalytic activity towards the ORR with a direct four-electron transfer pathway. The excellent ORR activity may be mainly attributed to high contents of graphitic N, pyridinic-N, and Co-N configurations. This study opens a new way to rationally design cheap and active ORR catalysts by using simple flat compound as a direct template.

**Electronic supplementary material:**

The online version of this article (doi:10.1186/s11671-016-1804-z) contains supplementary material, which is available to authorized users.

## Background

With the reduction of the storage volume of fossil fuels and the increasing emphasis on green environmental protection, people are trying to explore sustainable and non-polluting power sources. The development of high-efficiency fuel cells (or metal-air batteries) as the promising clean energy power generation technology has become the key solution to solve the problem of energy shortage and environment pollution around the world. However, the oxygen reduction reaction (ORR) at the cathode of these power sources has exhibited disadvantages of slow kinetics and the diversity of ORR pathway, which brings lots of negative effects to the battery system and further decreases the general performance of the battery [1, 2]. Platinum and its alloys are currently the best catalyst with ultrahigh ORR electrocatalytic activity [3], but we cannot perform the large-scale application because of their high price, low stability, and inferior tolerance to fuel molecules. Therefore, the search for cheap, highly active, and stable alternatives to the Pt catalyst can facilitate the commercialization of fuel cells.

Over the past few decades, many types of ORR non-Pt catalysts [4], including non-precious metal catalyst (NPMCs) [5], transition metal oxides (TMOs) [6], heteroatom-doped carbon material (HDCMs) [7], have been developed. Especially, nitrogen-doped carbon materials (NDCMs) have attracted much attention owing to their unique inner structure and catalytic properties, which are considered to be a new ORR catalyst for fuel cells [8]. In recent years, N-doped graphene (NG) [9], N-doped carbon nanotubes (N-CNTs) [10], N-doped carbon nanospheres (N-CNSs) [11], and other carbon nanomaterials have been effectively synthesized. Although they exhibit reasonable ORR catalytic activity and durability, the ORR catalytic mechanism and real active sites are unclear to this day. To be sure, heteroatoms (especially N atom) being introduced into the inner structure of carbon materials will improve the ORR catalytic activity in spite of doping methods (e.g., in situ and post-treatment doping). The enhancement of ORR activity in NDCMs is mainly due to the difference of bond length, valence electrons, and atomic size, which can result in the damage of electric neutrality of adjacent carbon atoms owing to the doping of nitrogen atoms [12, 13].

The main method of achieving higher ORR catalytic efficiency is to expose nitrogen-containing active sites for the ORR on the catalyst surface as soon as possible [12]. We previously formed a new class of ORR catalysts by pyrolysis of protein-rich biomass (e.g., animal biomass, blood protein, and enoki mushroom) modified on carbon materials (e.g., CNTs and CNSs) under innert atmosphere at high temperature [14–17]. We can find that the active sites exposed on the surface of CNTs or CNSs can largely enhance the ORR activity in alkaline and acidic media. Moreover, the addition of carbon support can hinder further birdnesting of decomposed products and promote the surface density of active sites for the ORR [15, 17]. Therefore, we report a new strategy to design a cobalt-nitrogen-doped carbon (Co-NC) composite nanocatalyst for oxygen reduction by using polyaniline (PANI) as nitrogen/carbon sources and program-controlled pyrolysis process at high temperatures. The montmorillonite can be used as a flat template in the synthesis of Co-NC catalyst. This catalyst exhibits an ORR electrocatalytic activity with a four-electron transfer pathway in both acidic and alkaline solutions.

## Methods

### Synthesis of Co-NC-900 Catalyst

**Fig. 1:**
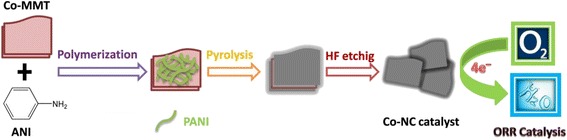
Schematic illustration for synthesis of the Co-NC catalyst

First, 2.0 g of montmorillonite (MMT) and 4.0 g of CoCl_2_ is added to a 50-ml beaker filled with 30 ml deionized water, and stirred for 24 h to fully exchange the Co^2+^ ion and Na^+^ ion at room temperature. After centrifugation at 2500 rpm, the obtained solid is dried at 80 °C to yield the Co-modified montmorillonite (Co-MMT). Subsequently, 0.40 g of Co-MMT and 0.40 g of aniline (ANI) are added into a 50-ml beaker filled with 20 ml deionized water, and regulated at pH = 2 with 0.1 mol l^–1^ HCl solution. Two grams of ammonium thiosulfate (APS) is further added into the above solution and stirred for 24 h to facilitate the polymerization of ANI at room temperature and form the precursor. The precursor is subsequently heat-treated at different temperatures (800, 900, and 1000 °C) for 2 h under Ar atmosphere. All produced samples were finally etched off in a 40 wt.% HF solution to prepare the Co-NC-X (*X* = 800, 900, or 1000) catalysts. The schematic illustration for preparation of Co-NC catalyst via using of Co-MMT flat template is indicated in Fig. 1. As a control, a similar method is performed to generate the NC-900 catalyst via directly using MMT flat template. Besides, the Co-PANI-900 was synthesized by direct polymerization of ANI monomers in the presence of the same Co^2+^ content without using of flat template.

### Structural and Electrochemical Characterizations

Field-emission scanning electron microscopy (FE-SEM) spectroscopy images were obtained by Hitachi UHR SU8020 (Japan). High-resolution transmission electron microscopy (HR-TEM) was carried out on a Zeiss LIBRA 200 FETEM instrument operating at 200 kV. X-ray photoelectron spectroscopy (XPS) was performed using a Kratos XSAM800 spectrometer equipped with an Al X-ray source (Al Kα, 1.4866 keV). The Koutecky–Levich plots were acquired by linear fitting of the reciprocal rotating speed versus reciprocal current density collected at various potentials. The electron transfer number was calculated from the following equation [12, 13]:

$$ 1/{j}_d=1/{j}_k+1/B{\omega}^{1/2} $$

$$ B=0.62n\mathrm{F}{\mathrm{C}}_{\mathrm{O}}{\mathrm{D}}_{\mathrm{O}}^{2/3}{\nu}^{-1/6}{\omega}^{1/2}, $$

where *n* is electron transfer numbers per oxygen molecule involved in the ORR, C_O_ is the O_2_ saturation concentration in the electrolyte, D_O_ is the O_2_ diffusion coefficient in the electrolyte, *ν* is the kinetic viscosity of the electrolyte, *ω* is the electrode rotation rate, and 0.62 is a constant when the rotation rate is expressed in rounds per minute.

Electrochemical data were collected on a Zahner Zennium-E electrochemical workstation (Germany) with a convential three-electrode cell at room temperature. A glass-carbon rotation disk electrode (GC-RDE, 4 mm in diameter, Model 636, Princeton Applied Research), a saturated calomel electrode (SCE), and a Pt foil with geometric area of 1 cm^2^ were used as working electrode, reference electrode, and counter electrode, respectively. All potentials are quoted versus a reversible hydrogen electrode (RHE) in this study. The preparation of working electrode was performed by a coating method. Typically, the obtained carbon catalyst was well-dispersed in the 0.5 wt.% Nafion/isopropanol solution. Five microliters of 10 mg ml^−1^ dispersion was transferred onto the GC-RDE surface and then naturally dried. The mass loading was estimated to be around 0.40 mg cm^−2^. A commercial Pt/C catalyst (20 wt.% Pt, E-ETK) on the GC-RDE surface was prepared in the same way, but its mass loading was kept at about 0.32 mg cm^−2^.

## Results and Discussion

### Catalyst Characterizations

**Fig. 2:**
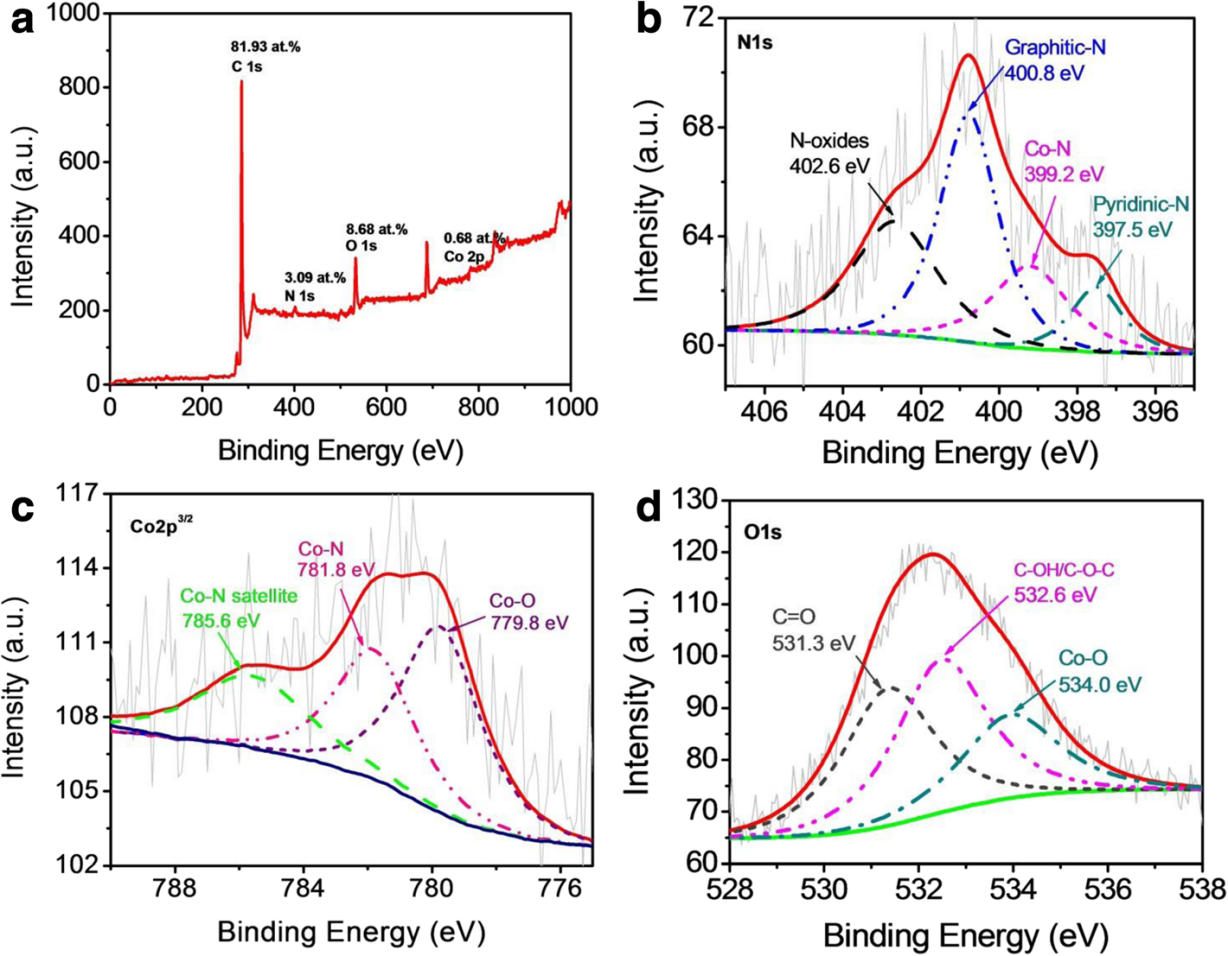
**a** XPS full-scan spectrum of Co-NC-900. Co 2p 3/2 (**b**), N1s (**c**), and O 1s (**d**) XPS spectra of Co-NC-900, respectively

**Fig. 3:**
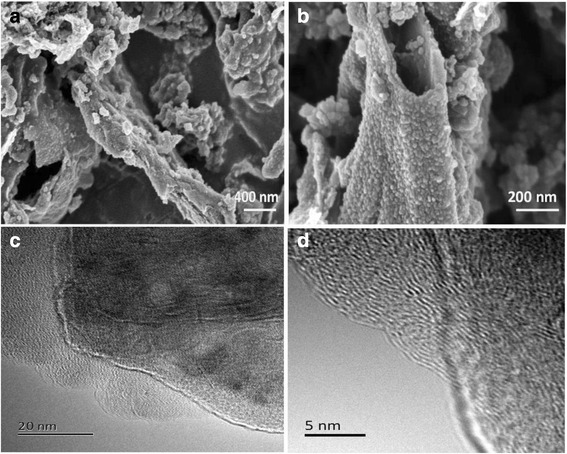
High-resolution SEM (**a**, **b**) and TEM (**c**, **d**) images of the Co-NC-900 catalyst

The element composition and chemical state of Co-NC-900 were analyzed by XPS technique. Carbon, nitrogen, oxygen, and cobalt elements were found in the catalyst at Fig. 2a with the relative percentage of 81.93, 3.09, 8.68, and 0.68 at.%, respectively. The occurrence of the nitrogen peak clearly indicate that nitrogen atoms were doped into the carbon skeleton structure of the Co-NC-900 catalyst. The N1s spectrum of Co-NC-900 can be deconvoluted into four peaks with binding energies of 397.5, 399.2, 400.8, and 402.6 eV (Fig. 2b), which can be assigned to pyridinic-N [12, 15], Co-N compounds [18], graphitic-N [16], and pyridinic-N-oxides [17], respectively. These results suggest that Co-N sites (19.4 at.%) and pyridinic-N species (10.3 at.%) can be formed in Co-NC-900, but the graphitic-N (40.9 at.% in total doped nitrogen) is still to dominate in all types of nitrogen functionalities. It has been acknowledged the graphitic-N plays a crucial role in oxygen reduction [19, 20], and that pyridinic-N and cobalt cations coordinated with N can also significantly increase ORR activity [21], these may be the main reason that the catalyst presents high ORR activity. For further confirming the presence of Co-N types of bond in N 1s spectra, we have done the analysis of Co2p^3/2^ spectrum for the same sample (Fig. 2c). In addition to the peak due to the form of cobalt oxide (779.8 eV) [22], there is a distinct peak due to the Co–Nx bond at 781.8 eV [18]. A significant amount of oxygen species are also included in the catalysts. The O 1s XPS spectrum of Co-NC-900 (Fig. 2d) is deconvoluted into three peaks with binding energies of 531.3, 532.6, and 534.0 eV, which can correspond to the C=O, C(aliphatic)–OH/C(aliphatic)–O–C(aliphatic), and metal-bounded oxygen, respectively. The comprehensive analysis of Co2p, N 1s, and O 1s spectra for the Co-NC-900 catalyst suggests that the cobalt atoms are in two forms (Co-N_*x*_ and Co-O). We have further characterized high-resolution SEM and TEM images of Co-NC-900, as displayed in Fig. 3. It is interestingly found that carbon nanosheets can be observed owing to the usage of MMT flat template for synthesis of Co-NC catalyst (see Fig. 3a, b). In addition, the Co-NC-900 catalyst mainly contains graphene-like nanostructures (Fig. 3c), and many crooked graphitic lattice fringes can be clearly observed at the exposed edge (Fig. 3d), suggesting a high degree of graphitization and an excellent graphitic structure.

### Oxygen Reduction Electrocatalyic Activity and Stability

**Fig. 4:**
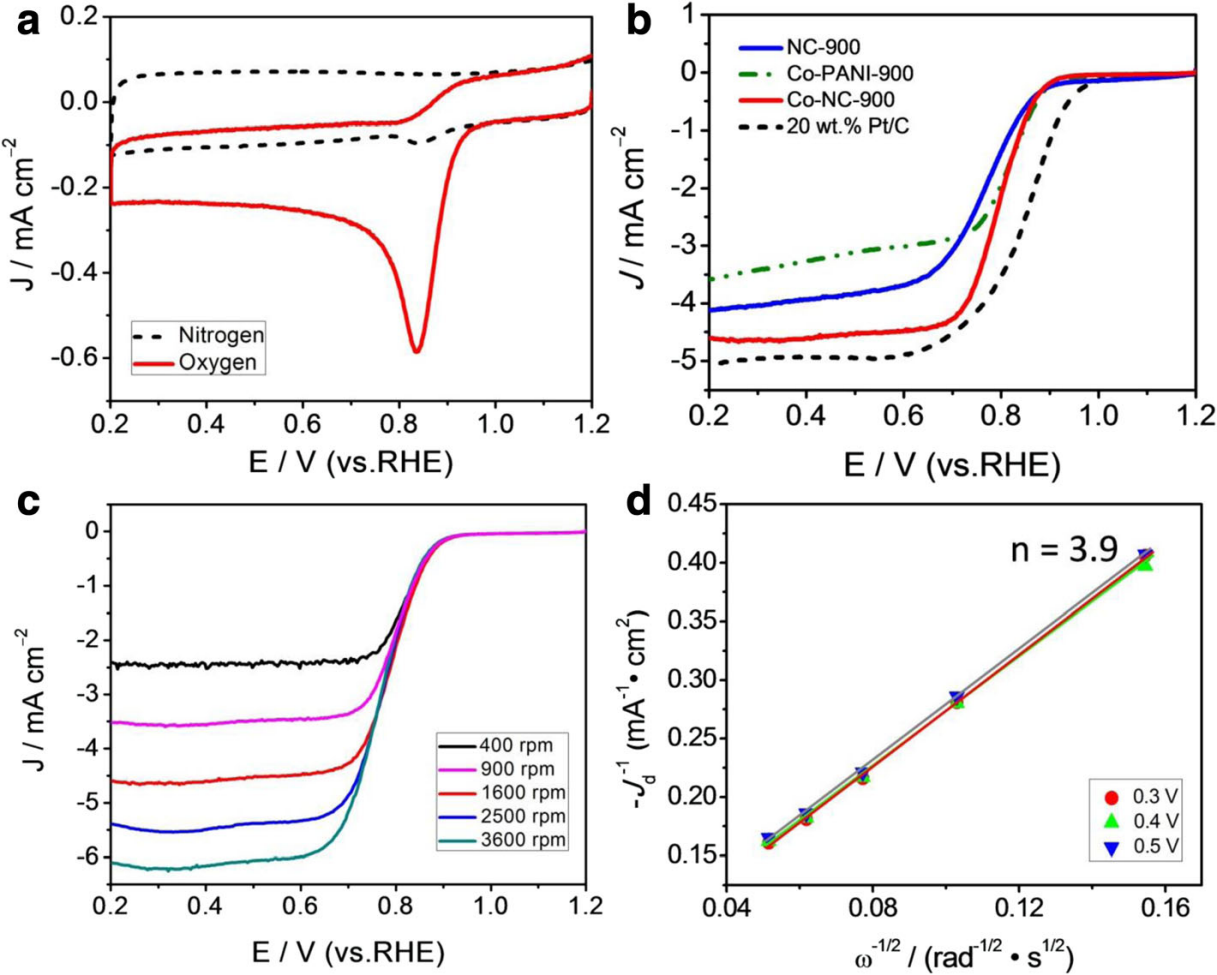
**a** CV curves of Co-NC-900 in 0.1 mol l^–1^ KOH solution saturated by nitrogen and oxygen. **b** LSV curves of Co-NC-900 in 0.1 mol l^–1^ KOH solution saturated by oxygen. **c** LSV curves of Co-NC-900 in 0.1 mol l^–1^ KOH solution at different rotation rates (400–3600 rpm). **d** K–L plots at 0.3–0.5 V; data are obtained from (**c**)

Cyclic voltammetry (CV) and linear sweep voltammetry (LSV) measurements in a conventional three-electrode system were performed to evaluate the electrocatalytic activity of the prepared catalyst towards (Co-NC-900, NC-900, and Co-PANI-900) the ORR in 0.1 mol l^–1^ KOH solution saturated by N_2_ or O_2_. Figure 4a clearly shows that the CV curve obtained in the condition of O_2_ saturation exhibits a significant reduction peak at about 0.84 V (versus RHE), but the CV curve obtained in N_2_-saturated solution is a featureless curve with a very weak reduction peak, which may be caused by unexhausted oxygen. It qualitatively suggests that the Co-NC-900 catalyst has an ORR electrocatalytic activity in alkaline medium. The RDE technique was further used to test the ORR activity and catalytic mechanism, as indicated in Fig. 4b–d. It can be observed that the ORR activity of Co-NC-900 catalyst with a half-wave potential (*E*_1/2_) of 0.80 V is obviously better than that of the NC-900 catalyst with a *E*_1/2_ of 0.77 V, showing that the addition of transition metal Co in the flat template can largely promote the formation of Co-N sites in Co-NC-900 catalyst, facilitating the effective improvement of ORR catalytic activity. Compared with the commercial 20 wt.% Pt/C catalyst, the *E*_1/2_ of Co-NC-900 is negatively shifted by about 50 mV and the *E*_1/2_ of NC-900 is negatively shifted by about 80 mV. However, the limited current density of Co-NC-900 is very close to that of 20 wt.% Pt/C catalyst. Besides, the limited current density of Co-NC-900 is far higher than that of Co-PANI-900, although their onset potentials are nearly equal, suggesting that the addition of flat template in the preparation of Co-NC catalyst can hinder the agglomeration of PANI under pyrolysis process and effectively expose more ORR active sites on the catalyst surface. The effects of heat-treatment temperature on the alkaline electrolyte ORR activity of Co-NC catalysts are also investigated in Additional file 1: Figure S1. By comprehensive analysis of ORR onset potentials and limited current densities of Co-NC catalysts obtained at different temperatures, it is definitely found that the optimal heat-treatment temperature is 900 °C for our prepared Co-NC catalysts, as higher or lower temperature will produce inferior ORR electrocatalytic activity in alkaline solution.

We further investigate the ORR electrocatalytic mechanism of Co-NC-900 using the RDE at different rotation rates (400–3600 rpm), as shown in Fig. 4c. As can be seen, an increase in the ORR current density with the rotation rate was observed at the Co-NC-900-catalyzed electrode. The good linearity of the Koutecky–Levich (K–L) plot (Fig. 4d) suggests the first-order dependence of the ORR kinetics at different potentials (0.3–0.5 V). The average ORR electron transfer number (*n*) is calculated to be about 3.9 at 0.3~0.5 V for Co-NC-900 and the average kinetic current density (*j*_k_) was calculated to be ~13.8 mA cm^–2^ for Co-NC-900, respectively, based on the slopes and intercepts of K–L plots obtained at 0.3–0.5 V (versus RHE). Our results indicate that the ORR on the Co-NC-900 catalyst proceeds mainly with four-electron reduction pathway (2 H_2_O + O_2_ + 4 e^−^ → 4 OH^−^), very similar to the ORR catalyzed by a state-of-the-art Pt/C catalyst measured in KOH solution [23]. More significantly, the ORR activity of Co-NC-900 is comparable to that of the best metal/nitrogen-doped carbon electrocatalysts and metal-free carbon-based electrocatalysts reported to date, in particular the catalysts derived from various nitrogen-containing organic molecules [14–17, 24–26]. Hence, we can reasonably conclude that the Co-NC-900 catalyst is a very promising candidate for the commercial Pt-based electrocatalyst in alkaline medium.

**Fig. 5:**
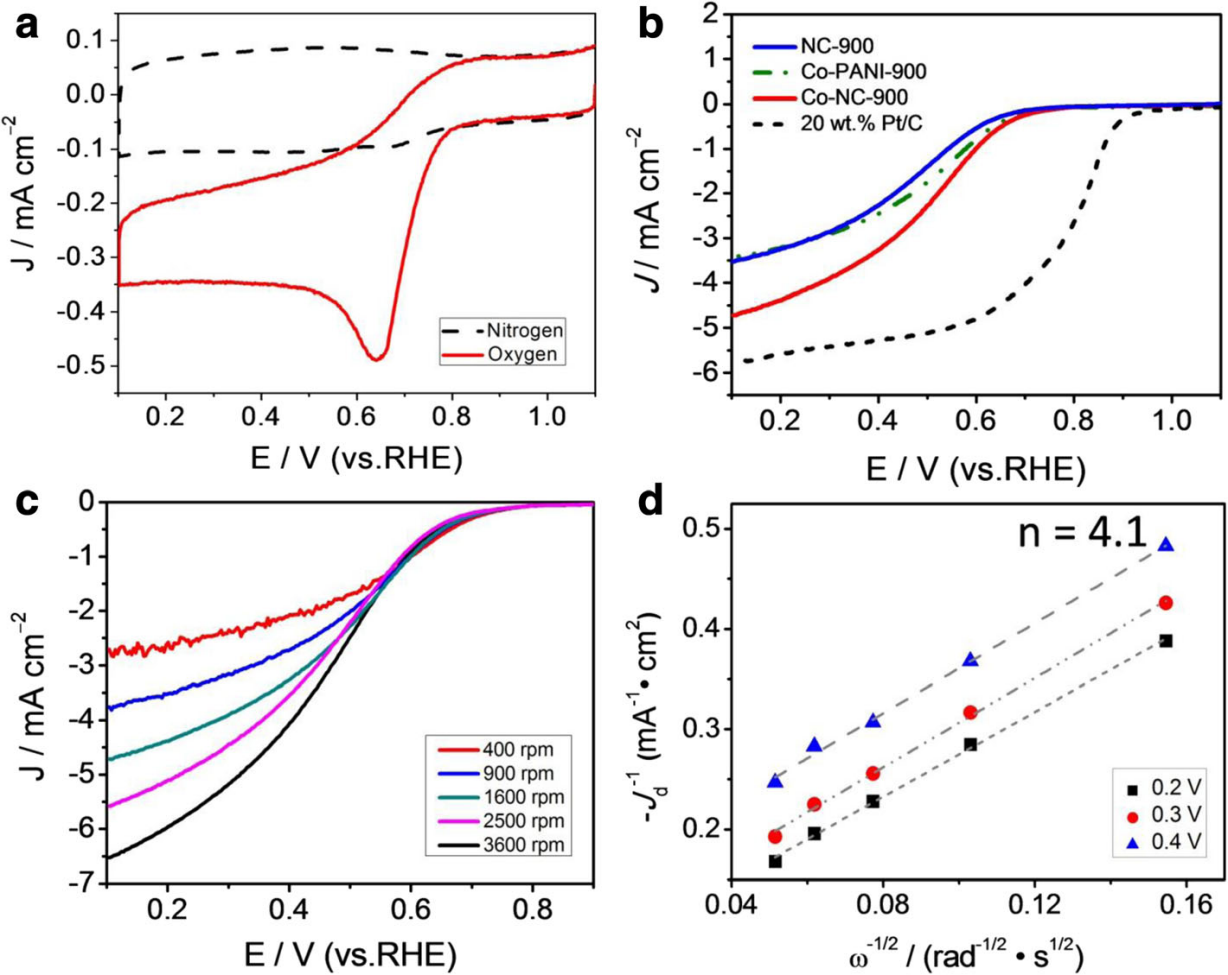
**a** CV curves of Co-NC-900 in 0.1 mol l^–1^ HClO_4_ solution saturated by nitrogen and oxygen. **b** LSV curves of in 0.1 mol l^–1^ HClO_4_ solution saturated by oxygen. **c** LSV curves of Co-NC-900 in 0.1 mol l^–1^ HClO_4_ solution at different rotation rates (400–3600 rpm). **d** K–L plots at 0.3–0.5 V; Data are obtained from (**c**)

The electrocatalytic performance of Co-NC-900, NC-900, and Co-PANI-900 towards the ORR was also tested in 0.1 mol l^–1^ HClO_4_ solution, as shown in Fig. 5a. A clear cathodic peak of ORR with a onset potential (*E*_ORR_) of 0.82 V and peak potential (*E*_p_) of 0.65 V is observed at the CV curve in O_2_-saturated 0.1 mol l^–1^ HClO_4_ solution, but no cathodic peak can be observed in N_2_-saturated 0.1 mol l^–1^ HClO_4_ solution, suggesting the electrocatalytic activity towards the ORR of Co-NC-900. The ORR activity was further studied by using RDE, as shown in Fig. 5b. It can be found that the ORR activity of Co-NC-900 catalyst is better than those of NC-900 and Co-PANI-900, but the *E*_1/2_ of Co-NC-900 catalyst about 300 mV lower than that of 20 wt.% Pt/C catalyst in HClO_4_ solution. Above results indicate that the prepared Co-NC-900 catalyst exhibits a higher ORR activity in alkaline medium than acidic medium. Additional file 1: Figure S2 indicates the influence of heat-treatment temperature on the ORR catalytic activity of Co-NC catalysts. A similar result can be obtained, which is that the optimal temperature is still 900 °C for the Co-NC catalyst, as higher or lower temperature will cause the ORR electrocatalytic activity to be worse in acidic solution.

To explain the ORR catalytic mechanism of the Co-NC-900 catalyst in acidic electrolyte, we also measured the ORR polarization curves in 0.1 mol l^–1^ HClO_4_ at different rotation speeds (400−3600 rpm), as displayed in Fig. 5c. The ORR current densities measured on Co-NC-900 increase with the increasing of RDE rotation rates. The good linearity of Koutecky–Levich (K–L) plots (Fig. 5d) and near parallelism of fitting lines synergistically show the first-order dependence of the ORR kinetics and similar electron transfer numbers for ORR at different potentials. The average electron transfer number (*n*) was calculated to be *ca.* 4.1 for Co-NC-900 and the average kinetic current density (*j*_k_) was calculated to be ~7.32 mA cm^–2^ for Co-NC-900, respectively, based on the slopes and intercepts of K–L plots obtained at 0.2–0.4 V (versus RHE). Hence, the ORR on Co-NC-900 proceeds with a direct four-electron reduction pathway (O_2_ + 4 H^+^ + 4e^–^ → 2H_2_O), very similar to the ORR catalyzed by the commercial Pt/C catalyst in 0.1 mol l^–1^ HClO_4_ solution [23].

**Fig. 6:**
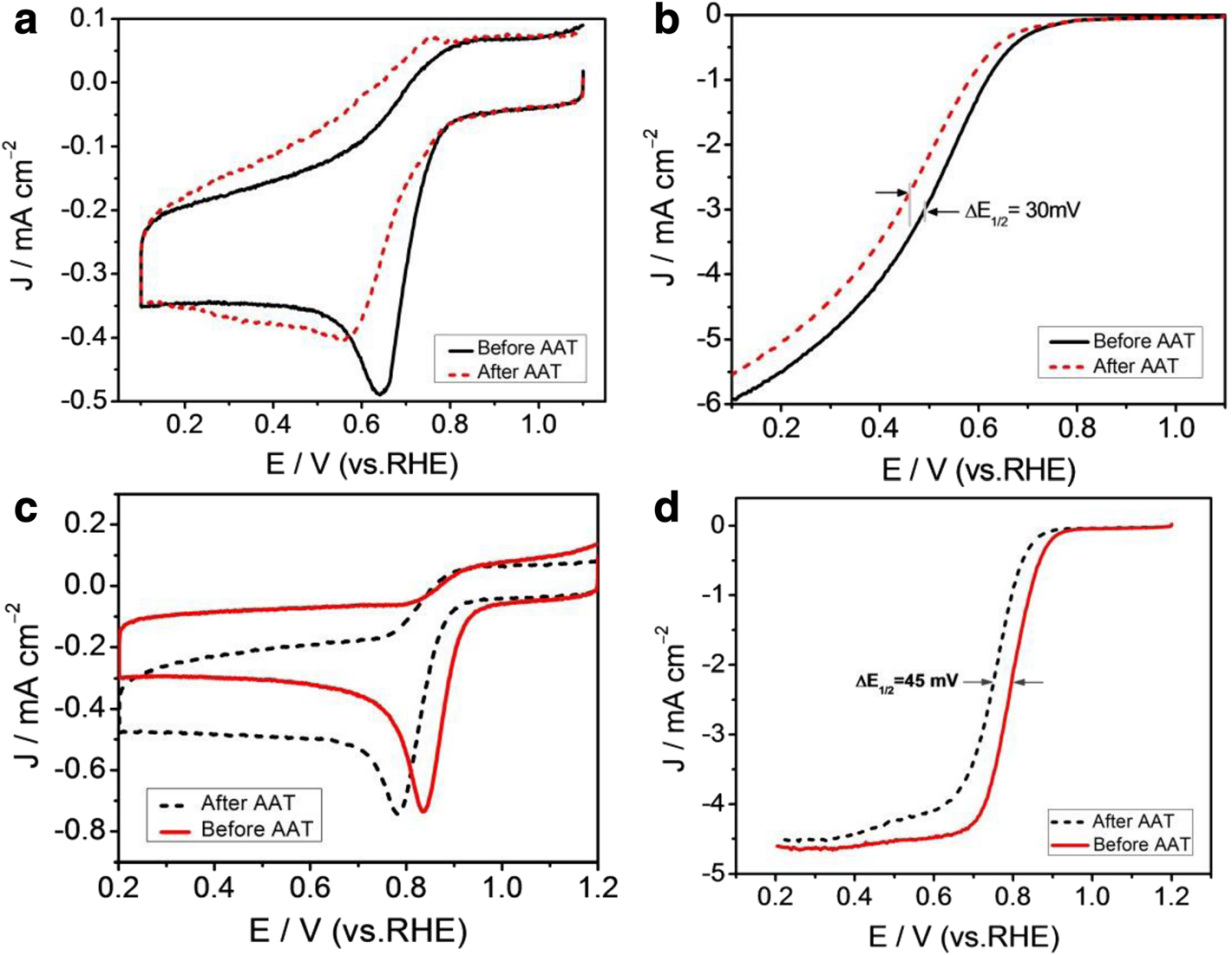
**a** CV curves of Co-NC-900 before and after AAT in O_2_-saturated 0.1 mol l^–1^ HClO_4_. **b** LSV curves of Co-NC-900 before and after AAT in O_2_-saturated 0.1 mol l^–1^ HClO_4_. **c** CV curves of Co-NC-900 before and after AAT in O_2_-saturated 0.1 mol l^–1^ KOH. **d** LSV curves of Co-NC-900 before and after AAT in O_2_-saturated 0.1 mol l^–1^ KOH

The electrochemical stability of Co-NC-900 in alkaline and acidic media is one of the most important reasons for assessing whether or not it can be applied to actual fuel cells. We have carried out an accelerated aging test (AAT) in O_2_-saturated 0.1 mol l^–1^ HClO_4_ or 0.1 mol l^–1^ KOH solution by continuous scanning for 6000 cycles at 50 mV s^–1^. After doing this, the ORR catalytic activity was measured by CV and LSV methods. It can be found that ORR peak potentials obtained at Co-NC-900 are negatively shifted after AAT in both HClO_4_ and KOH electrolytes, as shown in Fig. 6a, c. The LSV curves (Fig. 6b, d) clearly indicate that the ORR half-wave potentials of Co-NC-900 are obviously decreased about 30 mV in HClO_4_ solution and 45 mV in KOH solution, respectively. However, the decrease of limited current density is more obvious in HClO_4_ solution compared to that in KOH solution. Results from cycling stability tests show that the Co-NC-900 catalyst may be more suitable for application in alkaline electrolytes although the stability of Co-NC-900 is only comparable to that of Pt/C catalysts [27].

## Conclusions

Herein, we report a simple and new method to design a Co-NC catalyst by using a Co-modified montmorillonite as a flat template and using polyaniline as a single precursor of carbon and nitrogen, which can avoid the usage of complex chemicals substances in the synthetic process. The use of flat template can hinder the agglomeration of polyaniline during pyrolysis process and optimize the N-rich active site density on the surface owing to the intrinsic characteristics of Co-MMT template, resulting in the improvement of the ORR electrocatalytic activity in acidic and alkaline media. This study shows that under the condition of controlled temperatures, the Co-MMT template-assisted conversion of polyaniline is feasible to prepared a series of high-performance Co-NC catalysts for electrochemical reactions.

## Additional file

Additional file 1: Figure S1. (a) CV and (b) LSV curves of Co-NC catalysts in O_2_-saturated 0.1 mol l^–1^ KOH solution. **Figure S2.** (a) CV and (b) LSV curves of Co-NCcatalysts in O_2_-saturated 0.1 mol l^–1^ HClO_4_ solution. (DOCX 221 kb)
