# Supplementary material for: The Oxygen Reduction Electrocatalytic Activity of Cobalt and Nitrogen Co-doped Carbon Nanocatalyst Synthesized by a Flat Template
Source: Nanoscale Res Lett. 2017 Feb 22;12:144. doi: 10.1186/s11671-016-1804-z (PMC5321638; doi:10.1186/s11671-016-1804-z)
Supplement: Additional file 1: Figure S1. — (a) CV and (b) LSV curves of Co-NC catalysts in O2-saturated 0.1 mol l–1 KOH solution. Figure S2. (a) CV and (b) LSV curves of Co-NCcatalysts in O2-saturated 0.1 mol l–1 HClO4 solution. (DOCX 221 kb) [file 11671_2016_1804_MOESM1_ESM.docx]

**Additional file 1**

The Oxygen Reduction Electrocatalytic Activity of Cobalt and Nitrogen Co-doped Carbon Nanocatalyst Synthesized by a Flat Template

*Chaozhong Guo^*†^, Youcheng Wu^†^, Zhongbin Li, Wenli Liao^*^, Lingtao Sun, Chao Wang, Bixia Wen, Yanrong Li, Changguo Chen^*^*

^†^These authors equally contributed to this work, and they were considered as co-first authors.

***Corresponding authors.**

E-mail: [guochaozhong1987@163.com](mailto:guochaozhong1987@163.com); liaowenli@cqwu.net; cgchen@cqu.edu.cn

a


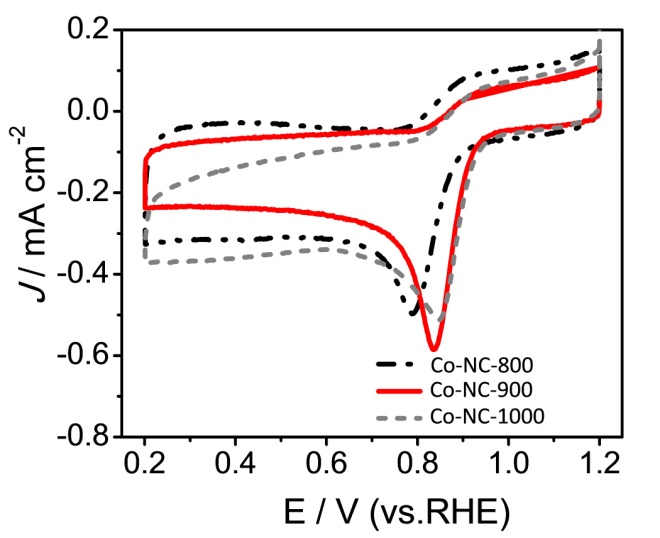


b

**Fig. S1**. (a) CV and (b) LSV curves of Co-NC-900 catalysts in O_2_-saturated 0.1 mol l^–1^ KOH solution.

b

a

**Fig. S2**. (a) CV and (b) LSV curves of Co-NC-900 catalysts in O_2_-saturated 0.1 mol l^–1^ HClO_4_ solution.
